# Ovulation induction in anovulatory southern white rhinoceros (*Ceratotherium simum simum*) without altrenogest

**DOI:** 10.1093/conphys/coz033

**Published:** 2019-06-24

**Authors:** Parker M Pennington, Kira L Marshall, Jonnie M Capiro, Rachel G Felton, Barbara S Durrant

**Affiliations:** 1 Reproductive Sciences, San Diego Zoo Institute for Conservation Research, Escondido CA, USA; 2 Lead Keeper, San Diego Zoo Safari Park, Rhino Rescue Center, Escondido CA, USA

**Keywords:** Anovulation, assisted reproductive technology (ART), GnRH, white rhinoceros

## Abstract

All species in the extant Rhinocerotidae family are experiencing increased threats in the wild, making captive populations essential genetic reservoirs for species survival. However, managed species face distinct challenges in captivity, resulting in populations that are not self-sustaining. Captive southern white rhinoceros (*Ceratotherium simum simum*) have low reproductive rates and presumed acyclicity is common among females. Although many females fail to ovulate, follicle growth may occur and ovulation can be hormonally induced. Female southern white rhino (*n* = 6), housed as a bachelorette group, were determined to be ovulatory (*n* = 1) or anovulatory (*n* = 5) by serial ultrasound and fecal progestagen analysis. When follicles reached pre-ovulatory size (~35 mm), females (*n* = 4) were induced to ovulate in 11 trials with a GnRH analog (4.5 mg, SucroMate™) via single intramuscular injection. Nine trials resulted in ovulation (81.8%), all between 36 and 48 hours post-treatment. Ovulations were confirmed by progestagen elevation above baseline coincident with visualization of a corpus luteum (CL). Luteal phases were characterized as short (<50 days) or long (≥50 days). Between short and long cycles, only the number of days of progestagen above baseline was significantly different (*P* < 0.05), while days with visible luteal structures was not significant (*P* = 0.11). Both cycle types were observed following both spontaneous and induced ovulations. Furthermore, we showed that longer cycle lengths do not necessarily indicate early pregnancy loss as none of the females were bred or inseminated during the study. While anovulation is common in the southern white rhino captive population, ovulation induction can be achieved efficiently and predictably for use in conjunction with artificial insemination or to facilitate natural breeding. This information will lead to more efficient use of assisted reproductive technologies to overcome reproductive challenges in this species and to generate genetically healthy captive populations as a hedge against extinction.

## Teaser

Lack of ovulation is common in captive southern white rhino females and contributes to poor reproductive success. We show that ovulation can be induced efficiently with exogenous hormones using a single treatment protocol. This information can lead to improved genetic management of captive populations acting as insurance against extinction.

## Introduction

The Sumatran (*Dicerorhinus sumatrensis*), Javan (*Rhinoceros sondaicus*) and black rhinoceros (*Diceros bicornis*) are designated as Critically Endangered. The greater one-horned (*Rhinoceros unicornis*) is listed as Vulnerable and the southern white (*Ceratotherium simum simum*) as Near Threatened by the IUCN (iucn.org, downloaded 07 June 2018). Survival in the wild is threatened by habitat loss and illegal poaching for their horns (Talukdar *et al.*, 2008; van Strien *et al.*, 2008a, b; Emslie, 2012a, b), rendering captive populations important genetic reservoirs for continued survival of these species. However, population growth is slow due to the lengthy 16-month gestation in all rhinoceros species. Additionally, each species has distinct reproductive characteristics (e.g. cycle length and pre-ovulatory follicle size; Roth, 2006). The southern white rhinoceros (SWR) experiences additional challenges in *ex situ* as a substantial number of captive females are diagnosed as ‘acyclic’ or ‘irregularly cyclic’ (Brown *et al*., 2001) and females born in captivity often experience infertility associated with high phytoestrogen diets (Tubbs *et al*., 2012, 2016). While the exact mechanism for acyclicity is unknown, it presents a specific barrier to overcome before assisted reproductive technique (ART) development can be pursued in earnest. ARTs such as ovarian control [including follicle stimulation, ovulation induction, corpus luteum (CL) disruption, etc.], artificial insemination (AI), ovum pickup and *in vitro* techniques can help overcome the challenges of rhinoceros reproduction in captivity. Development of these techniques relies heavily on a thorough understanding of female reproductive physiology (Roth, 2006; Hildebrandt *et al*., 2007; Hermes *et al*., 2009a, b; Roth *et al*., 2018). Despite substantial published hormone data describing SWR reproduction (Hindle *et al*., 1992; Schwarzenberger *et al*., 1998; Patton *et al*., 1999; Brown *et al*., 2001; Roth, 2006; Hermes *et al*., 2009b, 2012; van der Groot *et al*., 2013), relatively little longitudinal ultrasound data exist on estrous cycle characterization in this species (Radcliffe *et al*., 1997; Patton *et al*., 1999).

Estrous cycle parameters have been determined for the SWR and include two distinct cycle lengths of ~30 or 70 days (Radcliffe *et al*., 1997; Patton *et al*., 1999; Roth, 2006; Brown *et al*., 2001; Ververs *et al*., 2015). Since most studies on white rhinos have utilized only progesterone analysis to characterize estrous cycles (Schwarzenberger *et al*., 1998; Brown *et al*., 2001; Carlstead and Brown, 2005; Hermes *et al*., 2007), the acyclic designation may be incomplete. Although progesterone metabolite profiles indeed confirm that ovulation does not occur, the inability to accurately measure estrogen metabolites in white rhinos (Brown *et al*., 2001; Roth *et al*., 2018) leaves the question of cyclic follicle growth unanswered. Without concurrent ultrasound examination the possibility of ovarian activity cannot be excluded. For the purposes of this paper, we refer to females that exhibit follicular growth, but fail to ovulate, as anovulatory. The next steps in understanding anovulation (i.e. follicular growth without ovulation) include investigating the efficiency of ovulation induction with exogenous hormone treatment.

Gonadotropin releasing hormone (GnRH) is a common ovulation induction agent, and several commercially available analogs and formulations have been used in both greater one-horned and SWR (Stoops *et al*., 2004, 2016; Hildebrandt *et al*., 2007; Hermes *et al*., 2009b, 2012; Roth *et al*., 2018). However, published reports provide few details describing follicle growth, time from GnRH treatment to ovulation, follicle or reproductive tract characteristics or resulting luteal parameters. The omission of valuable information on ovarian events leading up to GnRH administration as well as those following induced ovulation (Hildebrandt *et al*., 2007; Hermes *et al*., 2009a, 2012) identified the need for additional data collection as well as investigation of a different treatment protocol.

Here we describe the utilization of an injectable GnRH analog (deslorelin acetate, SucroMate™), commonly used in domestic horse reproductive management, to induce ovulation in anovulatory SWR. We employed specific criteria for GnRH treatment and report resulting luteal phase parameters. Our aims were to (i) achieve ovulation from a novel protocol utilizing follicle growth and size as criteria for treatment, (ii) observe specific time of ovulation following treatment and (iii) describe differences between resulting cycle lengths.

As wild rhinoceros populations continue to decrease, captive populations will become critical resources for potential reintroduction efforts. Genetic diversity is a key element in a healthy population and can be a challenge to maintain in captivity. ARTs will enable more efficient use of genetic material, but they require a sound understanding of reproductive physiology as well as reproductive obstacles.

## Materials and methods

### Animals

This research was conducted as a part of a larger project and in accordance with animal use protocols approved by San Diego Zoo Global Institutional Animal Care and Use Committee (IACUC #15-009). Six SWR females, #2194, #2195, #2196, #2197, #2198 and #2199 (SB#s), were brought to the San Diego Safari Park Rhino Rescue Center (San Diego, CA, USA) in November 2015 from South Africa where they were semi-free ranging on three different conservancies. All females (*n* = 6) were wild-born and estimated ages ranged between 4 and 7 years old at the beginning of the study (Supplementary Material). Females were housed as a bachelorette herd at the time of data collection. One female #2197 came into captivity pregnant and delivered a stillborn calf in November, 2016. All females had access to a barn, cable yards, concrete-walled yards or exhibit (4 acres total) dependent upon daily management requirements. Each animal received 1.8–2.3 kg of commercial pellet individually, as well as supplemental timothy hay cubes, orchard grass hay and produce for training purposes. The group received up to two bales of Bermuda hay and free access to mineral blocks. Data were collected from September 2016 to August 2017 for all individuals except #2197 whose data were collected post-partum from December 2016 to August 2017.

### Serial ultrasound exams

Reproductive tract ultrasound exams were performed in a custom-designed chute allowing personnel protected access through a rear gate. The chute was equipped with stationary sides, such that rhino movement was limited but not restricted. Rhinos were trained with positive reinforcement and operant conditioning to stand voluntarily in the chute without sedation for transrectal ultrasound exams one to three times per week. Prior to entering the chute, individuals were behaviorally assessed. Keepers provided food items and verbal reinforcement during ultrasound sessions and all animal participation was voluntary. If the animal became agitated or restless the exam was terminated and she was released from the chute. Sessions typically lasted 10–30 min, including manual fecal voiding. A Sound LogiqE ultrasound unit with a 3.5–5 MHz convex probe was used for all exams. Each exam intended to image the cervix, uterine bifurcation, uterine horns and both ovaries. A probe extension allowed visibility of entire uterine horns and ovaries. Ovarian structures were classified, counted, measured and recorded.

### GnRH treatment trials

Treatment with a GnRH analog as a part of a criteria-specific protocol was evaluated. Deslorelin acetate (SucroMate™, 4.5 mg, 2.5 ml) was administered via single injection delivered to the neck by remote dart or by hand. Darts were immediately removed and all injections delivered the full dose. Injections were given when the following criteria were met: (i) follicle growth was confirmed by serial ultrasound exams and (ii) follicle diameter was ~35 mm. Females were examined via ultrasound 24, 36 and 48 hours post-injection. Following the 48-hour time point, animals were not examined again until their next twice weekly exam to confirm CL formation. Animal participation was voluntary and varied through the study period; therefore trial replications occurred more frequently in some animals than others.

### Fecal steroid extraction

Fecal samples were collected at least three times per week and stored at −20°C until processing prior to hormone analysis. Samples were lyophilized, pulverized and sifted through a 0.045-inch mesh screen to remove vegetation and debris. For all samples up to 5 May 2017, 0.1 g of sifted fecal material in a 16 × 100 mm glass tube was combined with 5 ml of 90% aqueous ethanol (EMD Millipore, Billerica, MA) and boiled at 80°C for 20 min. Samples were centrifuged (Thermo Scientific Sorvall Lengend XTR, Waltham, MA) for 10 min at 1000 x g at room temperature and the supernatant was recovered. Remaining fecal material was mixed with 5 ml 90% aqueous ethanol, pulse vortexed and centrifugation was repeated. Supernatants were combined, dried under air and resuspended in 1 ml ethanol.

For samples after 5 May 2017 0.2 g sifted fecal material in a 50 ml polypropylene tube was combined with 20 ml of 80% methanol (Fisher, Waltham, MA) in sterile water and vortexed at room temperature for 30 min (Fisher Scientific MultiTube Vortexer, Waltham, MA). Samples were centrifuged for 10 min at 4000 x g at room temperature (Thermo Scientific Sorvall Lengend XTR, Waltham, MA) and the supernatant was recovered.

Transition to a new fecal extraction method was initiated to decrease sample processing time and expedite analysis. The 80:20 methanol method was chosen based on reports by Palme *et al.* (1997, 2013) demonstrating this as the most effective method of hormone extraction. To ensure values were comparable between methods, extractions and radioimmunoassays (RIAs) for progestogens were performed with each method simultaneously (*n* = 196, distributed throughout 12 extractions and assays). Progestagen values did not differ between the two extraction methods (Pearson correlation *r* = 0.948, Paired *T*-test for significant difference *P* = 0.735). Methanol extraction efficiency measuring recovery of added tritiated progesterone (Perkin Elmer, Waltham, MA) was 82% ± 2.8%.

**Figure 1:**
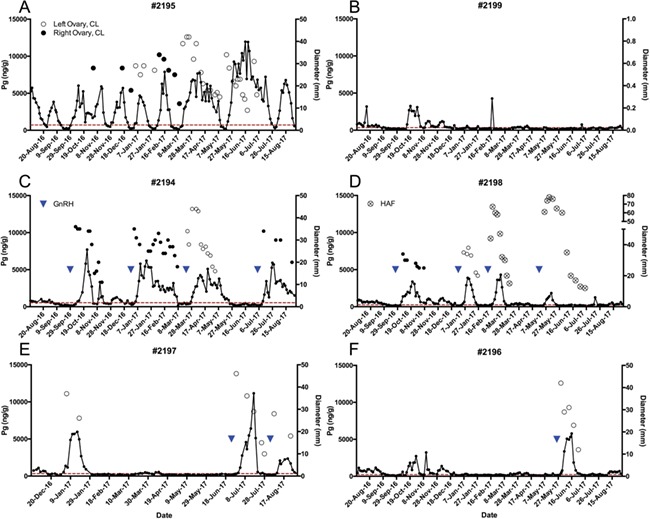
Progestagen profiles and CL diameters of individual southern white rhino females included in study. Individual progestagen profiles of southern white rhino study females. Circles (●, ○, ⊗) indicate mean diameter of CL following either spontaneous (#2195) or induced ovulations (#2194, #2196, #2197 and #2198). Blue triangles (▼) represent GnRH treatment. Ovulation was confirmed following treatment by elevation in progestagens above baseline (red dashed line) and CL visibility.

### RIA hormone analysis

Fecal progestagens were analyzed by RIA using 0.1 ml of a monoclonal antibody against 4-pregnen-3,20-dione (CL 425, Quidel, Santa Clara, CA; 1:43 000). This antibody cross-reacts with progesterone (100%), 4-pregnen-3a-ol-20-one (188%), 4-pregnen-3b-ol-20-one (172%), 4-pregnen-11a-ol-3,20-dione (147%), 5a-pregnan-3b-ol,20-one (94%), 5a-pregnan-3a- ol,20-one (64%), 5a-pregnan-3,20-dione (55%), 5b-pregnan-3b-ol-20-one (12.5%), 5b-pregnan- 3,20-dione (8%), 4-pregnen-11b-ol-3,20-dione (2.7%) and 5b-pregnan-3a-ol-20-one (2.5%). Other hormones tested were shown to cross-react at less than 1%, including 5a-Pregnan- 3a, 20b-diol, pregnandiol, androstendione and corticosterone. Tritiated progesterone (NET381250UC, Perkin Elmer, Waltham MA) diluted to 10 000–14 000 cpm (16 667–23 333 dpm) per 0.1 ml with ~40% total binding was used as the assay competitor against progesterone standards (7–1000 pg, Sigma, St Louis, MO).

Fecal extract dilutions were determined by parallelism and adjusted as necessary to ensure optimal assay antibody binding (20–85%). Two replicates of each sample extract were brought to 0.5 ml in 0.1 M phosphate buffered saline (PBS) pH 7.0 with 1% gelatin before assay. Following an overnight incubation at 4°C, addition of 0.25 ml charcoal–dextran solution [char–dex; 6.25 g charcoal (Fisher Scientific, Waltham, MA) and 0.625 g dextran (Sigma, St. Louis, MO) in 1 l PBS without gelatin] terminated competitive reactions. Char-dex-treated samples were incubated at 4°C for 30 min to separate bound from free hormone. Samples were then centrifuged at 4°C at 2000 x g for 15 min and supernatant was decanted into scintillation vials. Scintillation fluid (4 ml, MP Biomedicals, Santa Ana, CA) was added and samples were counted in a Beckman liquid scintillation counter (LS6500) for 1 min.

The assay was validated with a parallelism of serially diluted fecal neat and a progesterone standard curve (*r* = 0.986) and an accuracy test determined by recovery of known quantities of progesterone standards added to a pool of fecal extract (*R*^2^ = 0.986). Assay sensitivity was 7.55 pg/tube. Average intra-assay coefficient of variation was 8.59% and inter-assay coefficients of variation were 4.67% for low controls (average 45.5 pg/tube) and 3.52% for high controls (average 206.7 pg/tube). The RIA was also verified with reverse phase high performance liquid chromatography (Waters Nova-Pak Reverse Phase C18 4 mm 3.9 × 150 mm column, Milford, MA) to demonstrate progestagen detection.

### Statistical analysis

When ovulation was not induced, dominant follicle growth and subsequent regression coincident with no substantial progestagen rise above baseline was recorded as an anovulatory cycle. Baseline progestagen values were determined for each individual by an iterative process in which values higher than the mean plus 2 SD were excluded. The mean was then recalculated and the value exclusion process was repeated until no values were higher than the mean plus 2 SD (Brown *et al*., 2001). Cycle length was determined to be either short (<50 days) or long (≥50 days) by calculating the length of time progestagens were elevated above baseline following ovulation induction (female rhinos #2194, #2196, #2197 and #2198) or observed spontaneous ovulation (#2195). The 50-day cutoff was chosen because it falls evenly between previously determined cycle lengths of 30 or 70 days (Radcliffe *et al*., 1997; Patton *et al*., 1999; Brown *et al*., 2001; Roth, 2006; Ververs *et al.*, 2015) and has been used previously to determine long cycle lengths in SWR (Brown *et al*., 2001). Cycle parameters were as follows: number of days from observed ovulation to progestagen rise above baseline, number of days progestagens were elevated above baseline, maximum progestagen concentration (ng/g), number of days the CL was visible and maximum observed luteal structure size (mm). Cycle length and parameters are inclusive of days that were not sampled in order to calculate longest/largest possible time frames. All statistical analyses were performed using R studio (version 1.1.383). Welch’s *t*-test was used to determine significant differences between cycle parameters and cycle designations (‘short’ or ‘long’) as well as effect of treatment on follicle outcome (‘ovulation’ or ‘regression’; *stats* package, R Development Core Team, 2008). All data are presented as mean ± SD and considered significantly different at *P* < 0.05.

## Results

Ovarian follicular activity was apparent in all females examined during the study, although hormone analysis indicated anovulation in five of six females prior to treatment (Fig. 1). Notably, a single female (#2195) ovulated consistently, evidenced by regular fluctuations in fecal progestagen and single corpora lutea that were not concurrently visible (i.e. visible corpora lutea did not overlap temporally; Fig. 1A). This female exhibited both short (*n* = 5) and long (*n* = 3) cycles. Progestagens remained above baseline 22.0 ± 3.2 days (range, 19–26) in short cycles and 59.0 ± 9.5 days (range, 52–70) in long cycles (Table 1). Additional ultrasonographic information is not available as this female was only examined once per week. No ovulation inductions were performed on this individual.

**Table 1:** Endocrine and ultrasound parameters in ovulatory SWR female #2195

	Pg > baseline (days)^#^	CL visible (days)*	Max Pg (ng/g)	Max Pg after > baseline (days)	Max luteal size (mm)
short (*n* = 5)	22.0 ± 3.2	29.0 ± 1.4	5778.6 ± 1610.8	11.6 ± 4.0	31.5 ± 3.5
long (*n* = 3)	59.0 ± 9.5	50.0 ± 7.1	8577.7 ± 3050.7	21.3 ± 9.1	38.0 ± 5.7

mean ± SD;

Pg, progestagen.

^#^ *P* < 0.01.

^*^ *P* = 0.14.

**Figure 2:**
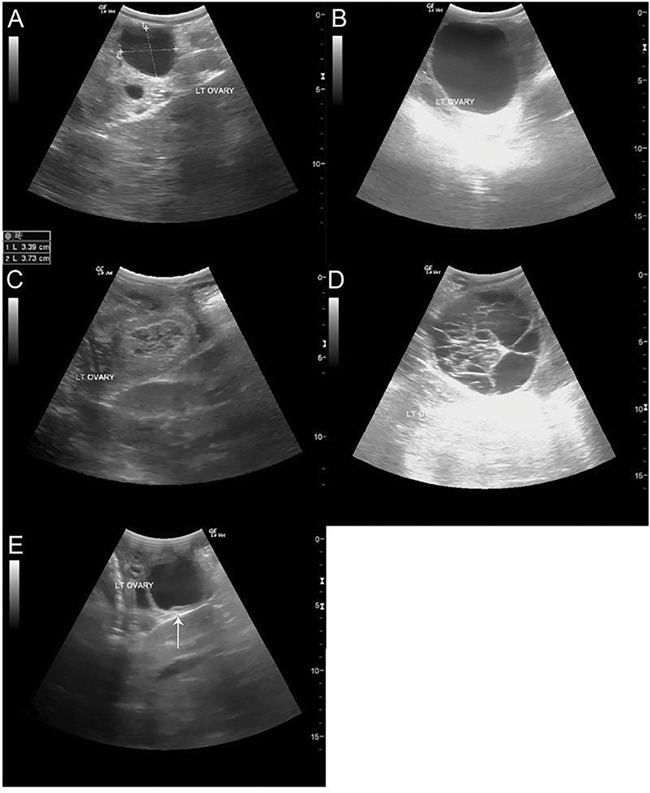
Ultrasound images of follicle types observed during study period. Representative images of follicle types observed during study period. (A) Dominant follicle that was treated with GnRH and resulted in ovulation. (B) Dominant follicle that was not treated and grew to ~60 mm, then regressed without ovulating. (C) CL following GnRH treatment. (D) HAF that formed following GnRH treatment with failure to ovulate. (E) Unstimulated follicle displaying a visual indicator of atresia, arrow indicates the lifting granulosa cell layer, which subsequently regressed without ovulating.

Two females (#2194 and #2198; Fig. 1C and D) showed no signs of ovulation prior to induction trials. Two females (#2196 and #2199; Fig. 1F and B) experienced brief elevations in progestagen without treatment, but no CL was visualized concurrent with progestagen rise and were therefore not considered to be ovulatory. Finally, #2197 came to the Safari Park pregnant and calved on 13 November 2016. She displayed a single ovulation ~45 days post-partum as evidenced by visualization of a CL and progestagen rise above baseline, but failed to ovulate spontaneously thereafter (Fig. 1E). During anovulatory cycles in all females except #2195, follicles grew to pre-ovulatory sizes, but then regressed. Follicles typically developed on alternating ovaries cyclically and growth often occurred during regression of a previously large follicle. Follicles appeared clear, without particulate infiltration or webbing (Fig. 2A, B and E). When follicles reached maximal size, visible indicators of regression were apparent such as separation of the granulosa cell layer from the follicle wall and the follicle wall becoming irregular or thickened (Fig. 2E). Anovulation was confirmed when fecal progestagens remained below baseline and there was no visible CL. Four females were induced to ovulate with GnRH treatment (#2194, #2196, #2197 and #2198), while one female (#2199) was not treated and did not form any luteal structures during the study period (Fig. 1B). Following ovulation induction and subsequent CL regression, the next dominant follicle was allowed to proceed without treatment. These unstimulated dominant follicles (*n* = 6) reached a maximal diameter (43.8 ± 6.1 mm; range, 37–54 mm), followed by regression and growth of another follicle that was subsequently induced to ovulate.

**Figure 3:**
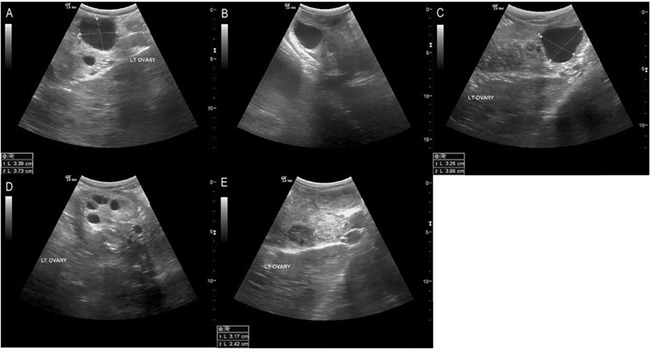
Ultrasound images of follicle progression through GnRH treatment. Representative ultrasound images of follicle progression at GnRH treatment through subsequent exams at 24, 36 and 48 hours post-administration, and CL formation. (A) Follicle on the day of GnRH treatment; (B) 24 hours post-treatment; (C) 36 hours post-treatment, note the shape change; (D) 48 hours post-treatment, the absence of the follicle indicative of ovulation. (E) CL evident following treatment.

**Figure 4:**
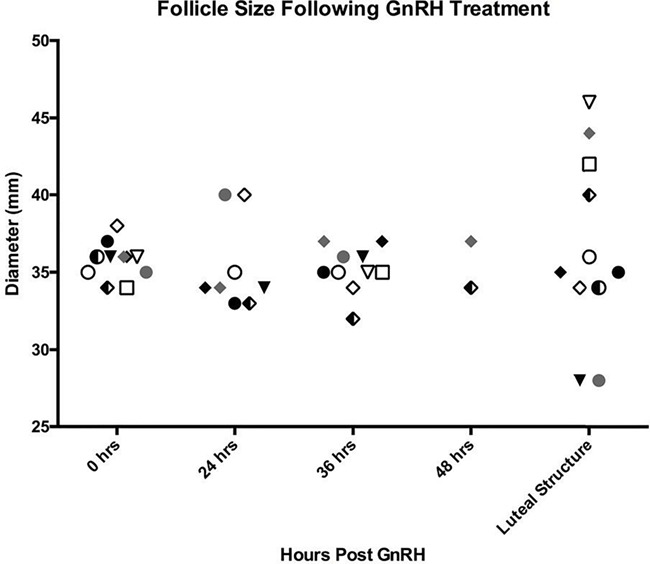
Follicle and luteal structure size following GnRH treatment. Follicle and luteal structure size post-GnRH plotted by individual for each treatment. Each shape represents an individual and color indicates treatment number. Circle (●) represents #2194, square (■) represents #2196, triangle (▼) represents #2197 and diamond (◆) represents #2198. Open shapes indicate first treatment, solid the second, gray the third and divided the fourth. Gray and divided diamonds (◆, #2198) identify treatments that resulted in HAF formation and were therefore present at 48 hours post-treatment.

GnRH treatment resulted in formation of a luteal structure, whereas no action resulted in regression of the follicle (*P* < 0.001). GnRH treatment resulted in ovulation 9 of 11 times (81.8%) and hemorrhagic anovulatory follicle (HAF) formation twice (18.2%; Table 2, Fig. 4). Both HAFs formed in the same female (#2198) and all long cycles after treatment occurred in the same female (#2194). Follicle size at treatment (0 hours) ranged from 34 to 36 mm in diameter. Following treatment, the follicle was observed at 24 and 36 hours but had disappeared by 48 hours. Ovulation was confirmed by CL formation (Fig. 3). HAF formation was determined by the persistence of the follicle at 48 hours followed by a substantial increase in size, up to 76 mm diameter, and development of fibrous echogenic bands that quivered upon ballotment (Fig. 2D). Mean follicle sizes were 35 mm at 24 and 36 hours for ovulations as well as at 48 hours post-treatment in follicles that formed HAFs, with luteal tissue averaging 36 mm 4–9 days post-treatment (36.6 ± 5.9; Fig. 4). These measurements are inclusive of HAFs as they fell within confirmed CL size ranges up to 4 days post-ovulation. However, maximal sizes were observed 7 and 15 days post-injection for each HAF (Fig. 1D). Progestogen levels were attenuated in HAF cycles (maximum, 1622 ng/g).

**Table 2:** Endocrine and ultrasound parameters for SWR treated with GnRH

	Pg > baseline (days)^#^	Luteal structure visible (days)*	Injection to Pg > baseline (days)	Max Pg (ng/g)	Max Pg after > baseline (days)	Max luteal size (mm)
short (*n* = 8)	27.4 ± 7.6	37.1 ± 13.6	6.9 ± 1.9	4788.5 ± 2964.7	13.3 ± 3.4	CL, 37.4 ± 5.7
long (*n* = 3)	62.7 ± 5.5	55.7 ± 13.1	7.0 ± 0	5601.7 ± 445.5	19.0 ± 5.3	HAF, 72.5 ± 7.8

mean ± SD; OV, ovulation; Pg, progestagen.

^#^ *P* < 0.01.

^*^ *P* = 0.11.

Ovulation was confirmed following treatment by elevation of progestagen above baseline for each individual (Fig. 1A–F) and visualization of a CL (Fig. 2C). Progestagens were elevated following HAF formation, and these data are included as cycle parameters (Fig. 1D). Both short (*n* = 8) and long (*n* = 3) cycles resulted from GnRH treatment (Table 2). Duration of progestagen elevation (days) was significantly different (*P* < 0.001) between short and long cycles, while visibility of luteal structures was not significant (*P* = 0.11). In treated females, progestagens were elevated for 27.4 ± 7.6 and 62.7 ± 5.5 days (range, 19–40 and 57–68 days) and luteal structures were visible for 37.1 ± 13.6 and 55.7 ± 13.1 days (range, 24–67 and 41–66 days) for short and long cycles, respectively. Average time from confirmed ovulation to progestagen rise above baseline, maximum progestagen and time to reach maximum progestogen levels were not significantly different between short and long cycles (Table 2). Interestingly, time from treatment to progestagen rise above baseline (days) was very similar (~7 days) for both short and long cycles. Substantial differences were seen in maximal luteal size between CL (37.4 ± 5.7 mm) and HAF formation (72.5 ± 7.8 mm), although only two HAFs contribute to this measurement.

## Discussion

We investigated the efficacy of ovulation induction in anovulatory SWR. We show that a novel ovulation induction protocol, based on natural follicle growth in anovulatory females, results in ovulation efficiently and reliably.

Brown *et al*. (2001) found numerous acyclic, or irregularly cyclic females at multiple institutions by fecal progesterone analysis. It is possible, however, that the females determined to be acyclic in this study experienced follicular growth but were anovulatory. Follicular waves without ovulation have been observed in SWR (Hermes *et al*., 2006; Roth *et al*., 2018) and were demonstrated in this study. The terms used to describe animals that fail to ovulate may need clarification: we suggest that anovulation is a form of acyclicity, from the perspective that ovulation is the defining aspect of the cycle and therefore a necessary component. Acyclic animals, determined by progesterone analysis, may grow follicles in a cyclical pattern without ovulation and can be identified by serial ultrasonography. More complete information about an individual designated as acyclic may expand the designation to anovulatory indicating that management options like ARTs might salvage reproductive potential and avoid the presumption of infertility commonly associated with acyclicity.

Exogenous GnRH resulted in luteal formation following all treatments in this study, although two instances resulted in HAF formation rather than ovulation. The injectable form of deslorelin acetate in oil was chosen for its straightforward application (single injection). This deslorelin preparation efficiently induces ovulation in horses, the model species for rhinos (Squires and Simon, 2011; Ferris *et al*., 2012). GnRH treatment in rhinos is intended to facilitate AI (Hildebrandt *et al*., 2007; Hermes *et al*., 2009b; Stoops *et al*., 2016); therefore ovulation efficiency is critical. We observed increased efficiency (81.8%) compared to a previous study (60.5%) with a similar sample size utilizing injectable GnRH in SWR (Hermes *et al*., 2012). In that study, a timed protocol used a 45-day treatment of altrenogest and induced ovulation 9 days after synthetic progestin withdrawal. Our dose of 4.5 mg GnRH was higher than the 3.0 mg dose utilized by Hermes *et al*. (2012) and we generated a narrowed window of ovulation between 36 and 48 hours post-injection. Furthermore, our time of observed response was longer than previously reported (Hildebrandt *et al.*, 2007; Hermes *et al.*, 2009b) in which ovulation was reported within 24 hours of GnRH administration. We tailored treatment to the individual animal and minimum follicle size, similar to the equine model (Squires and Simon, 2011; Ferris *et al*., 2012), rather than treat on an expected timed response, like the cattle model (Lamb *et al*., 2010). By customizing the treatment to each individual through serial ultrasonography and applying specific follicle growth and size criteria for treatment, rather than a timed approach, we observed rates of ovulation similar to those achieved in commercial equine practices (Finan *et al*., 2016; Squires and Simon, 2011). Precise ovulation timing and predictability expand the potential of AI and utility of cryopreserved sperm. We have also demonstrated that, similar to domestic horses, cycle lengths can be quite variable and a timed approach may not be suitable. Serial ultrasonography is beneficial to both domestic and exotic species reproductive management (Adams *et al.*, 1991) and was critical to the efficiency achieved here. We also observed a highly predictable time from injection to rise in fecal progestagen above baseline (~7 days) regardless of resulting luteal phase designation. With such efficiency and predictability achieved in ovulation induction, next steps include pairing with AI or developing other techniques such as embryo transfer.

Relying on hormone analysis alone to determine ovulation, as opposed to HAF formation, is cautioned against (Roth *et al*., 2018) and we observed a progestagen rise above baseline from HAF structures that mimicked luteal levels and duration. HAFs are commonly hormonally active and have been documented in all studied rhinoceros species (Roth *et al*., 2001, 2018; Stoops *et al*., 2004) and in horses (Ginther *et al*., 2006, 2008; Cuervo-Arango and Newcombe, 2012). Our observations of HAFs in only one female suggest certain individuals maybe pre-disposed to HAF formation. In horses, similar individual variability has also been observed (Ginther *et al*., 2006, 2008). Interestingly, although the HAFs observed in this study reached extremely large diameters, the duration of the HAF was designated as ‘short’ on both occasions, indicating the ability to resolve the structures quickly. Confirmation of ovulation, or lack thereof, was important as aged HAFs appeared similar to CL and highlights the need for frequent longitudinal ultrasonography when employing ARTs like AI.

We corroborate previous findings that white rhinos display two distinct estrous cycle lengths (Hindle *et al*., 1992; Radcliffe *et al*., 1997; Schwarzenberger *et al*., 1998; Patton *et al*., 1999; Brown *et al*., 2001). Length of time progestagens were elevated above baseline was significantly different between cycle designations, as expected. However, visibility of the resulting luteal structure was not significant between cycle designations and did not temporally correlate with progestagen elevation (data not shown). We suggest this may be due to the lower frequency of ultrasound data compared to that of fecal sampling. Fecal samples were collected at least three times per week while ultrasound data were collected one to three times per week with the exception of post-injection exams. The observation of luteal structures beyond progestagen elevation in short cycles, but not long cycles (Table 1), even when HAFs were excluded from analysis (data not shown) is interesting and warrants further investigation.

Long cycles have been purportedly caused by pathologies, such as pyometria, failed pregnancy or endometritis, and have been suggested to be abnormal (Radcliffe *et al*., 1997; Patton *et al*., 1999), while other studies indicate that the frequency of occurrence suggests that long cycles are normal (Schwarzenberger *et al*., 1998; Brown *et al*., 2001). Here we found that both cycle types can be exhibited within an individual (#2194 and #2195). Others (#2196, #2197 and #2198) displayed a single cycle type following ovulation induction, although the number of trials is too few per individual to conclude that these females do not exhibit both types. Longitudinal ultrasonography identified no uterine pathologies in any females throughout the study, and all cycles were non-conceptive with no potential for insemination, indicating that long cycles are not necessarily caused by pathology or failed pregnancy. Observation of both cycle types following ovulation induction in anovulatory females indicates that exogenous GnRH treatment produces luteal phases similar to spontaneous cycles. Although it is not known what dictates different cycle lengths, it appears both types are normal features of white rhino reproduction. The duration of the CL, and associated progestagen secretion that result in different luteal phase lengths, may be driven by the CL itself or the uterus. The conventional understanding for most mammals is that uterine prostaglandin secretion is down regulated by progestagen secretion from a CL and delays the onset of luteolysis. The functional capacity of the CL may be associated with the vascularity of the structure after ovulation and throughout the luteal phase as angiogenesis is a vital aspect of luteal formation and achievement of function (Fraser and Wulff, 2003; Tamanini and de Ambrogi, 2004). Increased vascularity, established at or after ovulation, may prolong the CL and enable sustained progestagen secretion, resulting in long cycles. Alternatively, because increased luteal blood flow has been shown to immediately precede prostaglandin secretion and luteolysis in cows (Miyamoto *et al*., 2005), perhaps highly vascularized rhino CLs may be more responsive to uterine prostaglandin secretion and result in short cycles. Future ultrasonographic studies utilizing color flow Doppler should focus on the extent of CL vascularity throughout the luteal phase to identify any relationships between blood flow and cycle length. Additionally, measurement of prostaglandin levels may provide more insight into the luteolytic process.

The criteria of follicle size (~35 mm) utilized in this study is larger than the previously documented pre-ovulatory follicle size (30 mm) for SWRs (Roth, 2006). Despite this, we observed a reliable response to GnRH. It is possible, though, that a range of effective sizes exists, as in horses (Ferris *et al*., 2012), and these limits have not been explored for rhinoceros species. We also propose that response to GnRH may vary in different housing situations, particularly if a male is present. Additionally, females that are determined to be ovulatory may have an altered response to GnRH as well, and the criteria for treatment may need to be amended. Nonetheless, since a large proportion of captive SWR females have been determined to be anovulatory via progesterone evaluation (Brown *et al*., 2001; Hermes *et al*., 2007), the next steps may be to ultrasonographically assess ovaries, monitor follicle growth and consider GnRH for induction of ovulation to facilitate breeding or AI.

Efficient use of ARTs would not only help overcome reproductive issues, like those common in captive SWR females, but also could improve animal welfare by obviating the need to transport animals between locations for breeding based on genetic matches. They may also circumvent behavioral incompatibilities experienced by an intended breeding pair. Furthermore, semen cryopreservation combined with AI or *in vitro* fertilization will maintain or improve the genetic diversity in a captive population by enabling breeding of genetic matches simultaneously at multiple locations. Information such as the data presented here will provide a route for more efficient use of other ARTs like AI and ET, which could ultimately modify the way captive populations are managed.

## Supplementary Material

Supplementary_Material_coz033

## References

[ref1] Adams GP, Plotka ED, Asa CS, Ginther OJ (1991) Feasibility of characterizing reproductive events in large non-domestic species by transrectal ultrasonic imaging. Zoo Biol 10: 247–259.

[ref3] Brown JL, Bellem AC, Fouraker M, Wildt DE, Roth TL (2001) Comparative analysis of gonadal and adrenal activity in the black and white rhinoceros in North America by noninvasive endocrine monitoring. Zoo Biol 20: 463–486.

[ref4] Carlstead K, Brown JL (2005) Relationships between patterns of fecal corticoid excretion and behavior, reproduction, and environmental factors in captive black (*Diceros bicornis*) and white (*Ceratotherium simum*) rhinoceros. Zoo Biol 24: 215–232.

[ref5] Cuervo-Arango J, Newcombe J (2012) Ultrasound characteristics of experimentally induced luteinized unruptured follicles (LUF) and naturally occurring hemorrhagic anovulatory follicles (HAF) in the mare. Theriogenology 77: 514–524. 21958645 10.1016/j.theriogenology.2011.08.026

[ref6] Emslie R (2012a) Ceratotherium simum. The IUCN Red List of Threatened Species 2012: eT4185A16980466 10.2305/IUCN.UK.2012.RLTS.T4185A16980466.en. Downloaded on 10 May 2019.

[ref7] Emslie R (2012b) Diceros bicornis. *The IUCN Red List of Threatened Species 2012: eT6557A16980917* 10.2305/IUCN.UK.2012.RLTS.T6557A16980917.en. Downloaded on 10 May 2019.

[ref8] Ferris R, Hatzel J, Lindholm A, Scofield DB, McCue PM (2012) Efficacy of deslorelin acetate (SucroMate™) on induction of ovulation in American quarter horse mares. J Equine Vet Sci 32: 285–288.

[ref9] Finan SA, Lamkin EL, McKinnon AO (2016) Comparative efficacy of BioRelease Deslorelin® injection for induction of ovulation in oestrus mares: a field study. Aust Vet J 94: 338–340. 27569838 10.1111/avj.12478

[ref10] Fraser HM, Wulff C (2003) Angiogenesis in the corpus luteum. Reprod Biol Endocrinol 1: 88 doi:https://doi.org/10.1186/1477-7827-1-88. 14613536 10.1186/1477-7827-1-88 PMC305342

[ref11] Ginther OJ, Gastal EL, Gastal MO, Beg MA (2006) Conversion of a viable preovuatlory follicle into a hemorrhagic anovulatory follicle in mares. Anim Reprod 3: 29–40.

[ref12] Ginther OJ, Gastal MO, Gastal EL, Jacob JC, Beg MA (2008) Induction of haemorrhagic anovulatory follicles in mares. Reprod Fertil Dev 20: 947–954. 19007559 10.1071/rd08136

[ref13] Hermes R et al. (2009a) Ovarian superstimulation, transrectal ultrasound-guided oocyte recovery, and IVF in rhinoceros. Theriogenology 72: 959–968. 19720394 10.1016/j.theriogenology.2009.06.014

[ref14] Hermes R, Goritz F, Saragusty J, Sos E, Molnar V, Schwarzenberger F, Hildebrandt TB (2009b) First successful artificial insemination with frozen-thawed semen in rhinoceros. Theriogenology 71: 393–399. 19007979 10.1016/j.theriogenology.2008.10.008

[ref15] Hermes R, Goritz F, Streich WJ, Hildebrandt TB (2007) Assisted reproduction in female rhinoceros and elephants – current status and future perspective. Reprod Domest Anim 42: 33–44. 17688600 10.1111/j.1439-0531.2007.00924.x

[ref16] Hermes R, Hildebrandt TB, Walzer C, Goritz F, Gray C, Niemuller C, Schwarzenberger F (2012) Estrus induction in white rhinoceros (*Ceratotherium simum*). Theriogenology 78: 1217–1223. 22898017 10.1016/j.theriogenology.2012.05.015

[ref17a] Hermes R, Hildebrandt TB, Walzer C, Göritz F, Patton ML, Silinski S, Anderson MJ, Reid CE, Wibbelt G, Tomasova K et al. (2006) The effect of long non-reproductive periods on the genital health in captive females white rhinoceroses *(Ceratotherium simum simum, C.s. cottoni)*. Theriogenology 65: 1492–1515. 16213012 10.1016/j.theriogenology.2005.09.002

[ref17] Hildebrandt TB, Hermes R, Walzer C, Sós E, Molnar V, Mezösi L, Schnorrenberg A, Silinski S, Streich J, Schwarzenberger F et al. (2007) Artificial insemination in the anoestrous and the postpartum white rhinoceros using GnRH analogue to induce ovulation. Theriogenology 67: 1473–1484. 17451805 10.1016/j.theriogenology.2007.03.005

[ref18] Hindle JE, Mostl E, Hodges JK (1992) Measurement of urinary oestrogens and 20a-dihydroprogesterone during ovarian cycles of black (*Diceros bicornis*) and white (*Ceratotherium simum*) rhinoceroses. J Reprod Fertil 94: 237–249. 1552485 10.1530/jrf.0.0940237

[ref19] Lamb GC, Dahlen CR, Larson JE, Marquezini G, Stevenson JS (2010) Control of the estrous cycle to improve fertility for fixed-time artificial insemination in beef cattle: a review. J Anim Sci 88: 181–192. 19783709 10.2527/jas.2009-2349

[ref20] Miyamoto A, Shirasuna K, Wijayagunawardane MPB, Watanabe S, Hayashi M, Yamamoto D, Matsui M, Acosta TJ (2005) Blood flow: a key regulatory component of corpus luteum function in the cow. Domest Anim Endocrinol 29: 329–339. 15888379 10.1016/j.domaniend.2005.03.011

[ref21] Palme R, Mostl E, Brem G, Schellander K, Bamberg E (1997) Faecal metabolites of infused 14C-progesterone in domestic livestock. Reprod Domest Anim 32: 1999–1206.

[ref22] Palme R, Touma C, Arias N, Dominchin MF, Lepschy M (2013) Steriod extraction: get the best out of faecal samples. Wien Tierarztl Monatsschr Vet Med Austria 100: 238–246.

[ref23] Patton M, Swaisgood RR, Czekala NM, White AM, Fetter GA, Montagne JP, Rieches RG, Lance VA (1999) Reproductive cycle length and pregnancy in the southern white rhinoceros (*Ceratotherium simum simum*) as determined by fecal pregnane analysis and observations of mating behavior. Zoo Biol 18: 111–127.

[ref24] R Development Core Team (2008) R: A language and environment for statistical computing In R Foundation for Statistical Computing, Vienna, Austria, ISBN 3-900051-07-0, http://www.R-project.org

[ref25] Radcliffe RW, Czekala NM, Osofsky SA (1997) Combined serial ultrasonography and fecal progestin analysis for reproductive evaluation of the female white rhinoceros (*Ceratotherium simum simum*): preliminary results. Zoo Biol 16: 445–456.

[ref26] Roth TL (2006) A review of the reproductive physiology of rhinoceros species in captivity. Int Zoo Yearb 40: 130–143.

[ref27] Roth TL, O’Brien JK, McRae MA, Bellem AC, Romo SJ, Kroll JL, Brown JL (2001) Ultrasound and endocrine evaluation of the ovarian cycle and early pregnancy in the Sumatran rhinoceros, *Dicerohinus sumatrensis*. Reproduction 121: 139–149. 11226037 10.1530/rep.0.1210139

[ref28] Roth TL, Schook MW, Stoops MA (2018) Monitoring and controlling ovarian function in the rhinoceros. Theriogenology 109: 48–57. 29249327 10.1016/j.theriogenology.2017.12.007

[ref29] Schwarzenberger F, Walzer C, Tomasova K, Vahala J, Meister J, Goodrowe KL, Zima J, Straub F, Lynch M (1998) Fecal progesterone metabolite analysis for non-invasive monitoring of reproductive function in the white rhinoceros (*Ceratotherium simum simum*). Anim Reprod Sci 53: 1723–1790. 10.1016/s0378-4320(98)00112-2 9835375

[ref30] Squires EL, Simon BW (2011) Evaluation of a new sustained-release deslorelin acetate for induction of ovulation in mares. AAEP Proceedings 57: 53–54.

[ref31] Stoops MA, Campbell MK, DeChant CJ, Hauser J, Kottwitz J, Pairan RD, Shaffstall W, Volle K, Roth TL (2016) Enhancing captive Indian rhinoceros genetics via artificial insemination of cryopreserved sperm. Anim Reprod Sci 172: 60–75. 27449405 10.1016/j.anireprosci.2016.07.003

[ref32] Stoops MA, Pairan RD, Roth TL (2004) Follicular, endocrine, and behavioral dynamics of the Indian rhinoceros (*Rhinoceros unicornis*) oestrous cycle. Reproduction 128: 843–856. 15579602 10.1530/rep.1.00328

[ref33] Tamanini C, De Ambrogi M (2004) Angiogenesis in developing follicle and corpus luteum. Reprod Domest Anim 39: 206–216. 15225273 10.1111/j.1439-0531.2004.00505.x

[ref34] Talukdar BK, Emslie R, Bist SS, Choudhury A, Ellis S, Bonal BS, Malakar MC, Talukdar BN, Barua M (2008) Rhinoceros unicornis. *The IUCN Red List of Threatened Species 2008: eT19496A8928657* 10.2305/IUCN.UK.2008.RLTS.T19496A8928657.en. Downloaded on 10 May 2019.

[ref35] Tubbs C, Hartig P, Cardon M, Varga N, Milnes M (2012) Activation of southern white rhinoceros (*Ceratotherium simum simum*) estrogen receptors by phytoestrogens: potential role in the reproductive failure of captive-born females. Endocrinology 3: 1444–1452. 10.1210/en.2011-1962 PMC3281539 22253418

[ref36] Tubbs C, Moley LA, Ivy JA, Metrione LC, LaClaire S, Felton RG, Durrant BS, Milnes MR (2016) Estrogenicity of captive southern white rhinoceros diets and their association with fertility. Gen Comp Endocrinol 238: 32–38. 27167501 10.1016/j.ygcen.2016.05.004

[ref37] van der Groot AC, Dalerum F, Ganswindt A, Martin GB, Millar RP, Paris MCJ (2013) Faecal progestogen profiles in wild southern white rhinoceros (*Ceratotherium simum simum*). Afr Zool 48: 143–151.

[ref38] van Strien NJ, Manullang B, Sectionov Isnan W, Khan MKM, Sumardja E, Ellis S, Han KH, Boeadi Payne J, Bradley Martin E (2008a) Dicerorhinus sumatrensis. *The IUCN Red List of Threatened Species 2008: eT6553A12787457* 10.2305/IUCN.UK.2008.RLTS.T6553A12787457.en. Downloaded on 10 May 2019.

[ref39] van Strien NJ, Steinmetz R, Manullang B, Sectionov Han KH, Isnan W, Rookmaaker K, Sumardja E, Khan MKM, Ellis S (2008b) Rhinoceros sondaicus. *The IUCN Red List of Threatened Species 2008: eT19495A8925965* 10.2305/IUCN.UK.2008.RLTS.T19495A8925965.en. Downloaded on 10 May 2019.

[ref40] Ververs C, van Zijl Lanhou M, Govaere J, Van Som A (2015) Features of reproduction and assisted reproduction in the white (*Ceratotherium simum*) and black (*Diceros bicornis*) rhinoceros. Vlaams Diergeneeskd Tijdschr 84: 175–184.

