# Supplementary material for: Ovulation induction in anovulatory southern white rhinoceros (Ceratotherium simum simum) without altrenogest
Source: Conserv Physiol. 2019 Jun 24;7(1):coz033. doi: 10.1093/conphys/coz033 (PMC6589769; doi:10.1093/conphys/coz033)
Supplement: Supplementary_Material_coz033 [file supplementary_material_coz033.docx]

Supplementary Table 1. Demographics of southern white rhino included in study.

| Individual  (SB#) | Estimated Age (years) | Reproductive Status | Data Collection | Number of  Treatments |
| --- | --- | --- | --- | --- |
| #2194 | 7 | Anovulatory | Sept 2016 – Aug 2017 | 4 |
| #2195 | 4 | Ovulatory | Sept 2016 – Aug 2017 | 0 |
| #2196 | 4 | Anovulatory | Sept 2016 – Aug 2017 | 1 |
| #2197* | 6 | Anovulatory | Dec 2016 – Aug 2017 | 2 |
| #2198 | 5 | Anovulatory | Sept 2016 – Aug 2017 | 0 |
| #2199 | 4 | Anovulatory | Sept 2016 – Aug 2017 | 4 |

*Parous female, calving date Nov 13, 2016; all other females assumed nulliparous

Supplementary Table 2. Endocrine and ultrasound parameters in ovulatory SWR female #2195.

| Individual | Pg >  baseline  (days) | CL visible  (days) | OV to Pg > baseline (days) | Max Pg (ng/g) | Max Pg after > baseline  (days) | Max luteal size (mm) | Luteal structure | Cycle designation |
| --- | --- | --- | --- | --- | --- | --- | --- | --- |
| #2195 | 19 | NA | NA | 3843 | 9 | NA | CL | short |
|  | 52 | NA | NA | 6028 | 11 | NA | CL | long |
|  | 26 | NA | NA | 5709 | 18 | NA | CL | short |
|  | 21 | 28 | NA | 4681 | 8 | 29 | CL | short |
|  | 19 | 30 | NA | 7882 | 10 | 34 | CL | short |
|  | 56 | 55 | NA | 7746 | 25 | 42 | CL | long |
|  | 70 | 45 | NA | 11957 | 28 | 34 | CL | long |
|  | 25 | NA | NA | 6776 | 13 | NA | CL | short |

CL = corpus luteum, Pg = progestagen

Supplementary Table 2. Endocrine and ultrasound parameters in ovulatory SWR females #2194, #2196, #2197, and #2198.

| Individual  (SB#) | Pg >  baseline (days) | Luteal structure visible (days) | Injection to Pg > baseline (days) | Max Pg (ng/g) | Max Pg after > baseline (days) | Max luteal size (mm) | Luteal structure | Cycle designation |
| --- | --- | --- | --- | --- | --- | --- | --- | --- |
| #2194 | 40 | 45 | 11 | 7712 | 16 | 36 | CL | short |
|  | 63 | 66 | 7 | 6211 | 17 | 35 | CL | long |
|  | 57 | 41 | 7 | 5101 | 25 | 44 | CL | long |
|  | 68 | 60 | 7 | 5926 | 15 | 34 | CL | long |
| #2196 | 31 | 32 | 5 | 5787 | 17 | 42 | CL | short |
| #2197 | 27 | 31 | 6 | 11153 | 17 | 46 | CL | short |
|  | 22 | 30 | 6 | 2353 | 12 | 28 | CL | short |
| #2198 | 36 | 38 | 8 | 3382 | 17 | 34 | CL | short |
|  | 23 | 24 | 6 | 3887 | 8 | 38 | CL | short |
|  | 21 | 30 | 6 | 4312 | 13 | 67 | HAF | short |
|  | 19 | 67 | 7 | 1622 | 11 | 78 | HAF | short |

CL = corpus luteum, Pg = progestogen, HAF = hemorrhagic anovulatory follicle
